# Supplementary material for: Collective modes and quantum effects in two-dimensional nanofluidic channels
Source: Faraday Discuss. 2023 Jun 28;249:162–80. doi: 10.1039/d3fd00115f (PMC10845119; doi:10.1039/d3fd00115f)
Supplement: FD-249-D3FD00115F-s001 [file FD-249-D3FD00115F-s001.pdf]

Electronic Supplementary Material (ESI) for Faraday Discussions.
This journal is © The Royal Society of Chemistry 2023

Collective modes and quantum effects in two-dimensional nanofluidic channels
Electronic Supplementary Information (ESI)

Baptiste Coquinot,[a,b,c] Maximilian Becker,[d] Roland R. Netz,[d] Lydéric Bocquet[a] and Nikita Kavokine[b,c]*
[a] *Laboratoire de Physique de l'École Normale Supérieure, ENS, Université PSL, CNRS, Sorbonne Université, Université Paris Cité, 24 rue Lhomond, 75005 Paris, France.*
[b] *Department of Molecular Spectroscopy, Max Planck Institute for Polymer Research, Ackermannweg 10, 55128 Mainz, Germany.*
[c] *Center for Computational Quantum Physics, Flatiron Institute, 162 5th Avenue, New York, NY 10010, USA.*
[d] *Fachbereich Physik, Freie Universitat Berlin, Arnimallee 14, 14195 Berlin, Germany.*

CONTENTS

I. Absence of overscreening with the confined response functions 1

II. Electrostatic interaction confinement 1
A. Model and solid's static response function 1
B. Interaction confinement for an ion and ion-ion electrostatic interaction 3
C. Interaction confinement for a dipole and dipole-dipole electrostatic interaction 3

III. Water response functions and simulations 4
A. Numerical methods for water simulations 4
B. Fluctuation-dissipation theorem 4
C. Evaluation of the time correlation function 4
D. Momentum dependence and position of the interface 5

References 5

I. ABSENCE OF OVERSCREENING WITH THE CONFINED RESPONSE FUNCTIONS

Since the real part of the surface response functions consists of positive numbers smaller than one, Eq. (16) of the main text allows the antisymmetric response function to become larger than 1. This is in particular the case at zero frequency when the imaginary parts vanish.

Physically, it is easy to understand that the symmetric response function should be smaller than the surface response function while its antisymmetric counterpart should be larger. Indeed, let us focus on the top solid, which responds to the sum of the (positive) external potential and the potential induced by the bottom solid. In the symmetric case the bottom solid also responds to a positive external potential and then induces a negative potential which is damped exponentially. However, the surviving part reaching the top solid is still negative and then reduces the effective external potential to which the top solid responds. The symmetric response is then smaller than the surface response because the external potential is screened by the bottom solid. On the contrary, in the antisymmetric case, the external potential is negative on the bottom solid which then responds by inducing a positive field. By the same process, the top solid now sees an effective external potential which is larger, generating a larger response. The antisymmetric response should then be larger than the surface response.

However, this cannot generate overscreening. Indeed, even if the antisymmetric response function is larger than 1, one should remember that the induced potential is not of the form of the external potential but of its "conjugate". The antisymmetric inner weight function is smaller than its outer counterpart: this solves the apparent problem. Let us formalise this intuition by computing the ratio of the induced field by the external field between the interfaces:

$$-\frac{\phi_{\mathrm{ind}}(z)}{\phi_{\mathrm{ext}}(z)} = g_{\mathrm{e}}^{\mathrm{x}}\frac{F_{\mathrm{i}}^{\mathrm{x}}(z)}{F_{\mathrm{o}}^{\mathrm{x}}(z)} = \frac{g_{\mathrm{o}}e^{-qh}}{1+\eta g_{\mathrm{o}}e^{-qh}}\frac{|e^{qz}+\eta e^{-qz}|}{e^{-q|z|}}. \quad (1)$$

where $\eta = \pm 1$ depending on whether we consider the symmetric or antisymmetric case. This ratio is maximal for $z = \pm h/2$ where it reaches

$$-\frac{\phi_{\mathrm{ind}}(\pm h/2)}{\phi_{\mathrm{ext}}(\pm h/2)} = g_{\mathrm{o}}\frac{1+\eta e^{-qh}}{1+\eta g_{\mathrm{o}}e^{-qh}} = 1 - \frac{1-g_{\mathrm{o}}}{1+\eta g_{\mathrm{o}}e^{-qh}} \leq 1. \quad (2)$$

Therefore, the induced potential is always smaller than the external potential, even when the confined response function is larger than 1. There is no overscreening.

As a consequence, the water static confined response function must fulfill the inequality $g_{\mathrm{w}}^{\mathrm{x}} \leq 1+\eta e^{-qh}$. In particular, the static antisymmetric response function goes to zero at $q \to 0$. For comparison, its surface response function has the limit $g_{\mathrm{w}}(\omega = 0, q \to 0) \to (\epsilon-1)/(\epsilon+1) \approx 0.98$ (see [1]).

II. ELECTROSTATIC INTERACTION CONFINEMENT

A. Model and solid's static response function

In this section, we focus on how confinement will modify the electrostatic interactions between charges inside a

* Contact: nikita.kavokine@mpip-mainz.mpg.de.

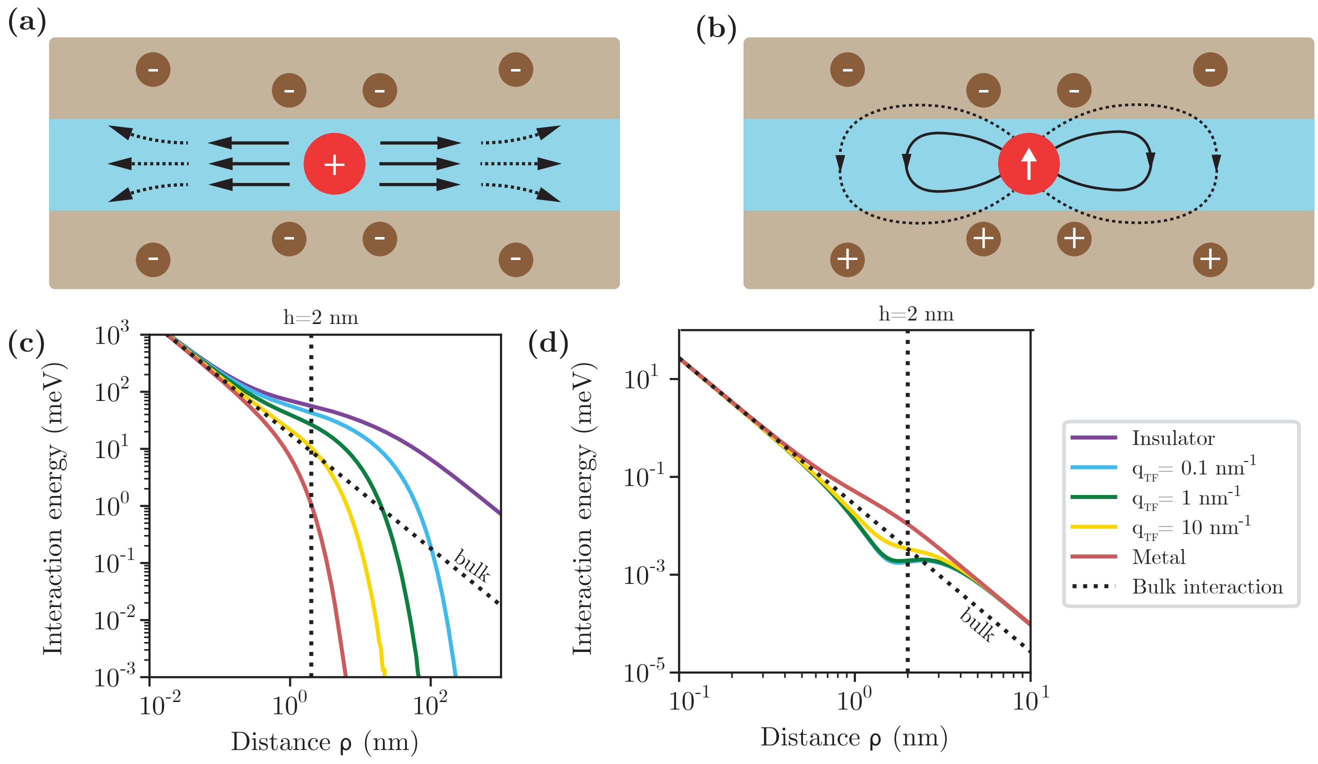

FIG. 1. a) Interaction confinement of an ion in the center of the channel: the generated electric field is partly confined in the channel by the surrounding solid which develop counter-charges (pictured in dark brown). This leads to a modified ion-ion interactions. b) Interaction confinement of an electrostatic dipole in the center of the channel: the generated electric field is partly confined in the channel by the surrounding solid which develop counter-charges (pictured in dark brown). This leads to a modified dipole-dipole interactions. c) Ion-ion interaction energy shift (normalised by the bulk interaction energy) for two ions at a distance ρ in bulk (black dashed line) and in a channel of confinement $h = 2$ nm. Different models of solid are used. d) Dipole-dipole interaction energy shift (normalised by the bulk interaction energy) for two dipoles in the z-direction at a distance ρ in bulk (black dashed line) and in a channel of confinement $h = 2$ nm. Different models of solid are used.

**Model**	**Geometry**	**Weight function**	
**Single interface**	Half-space	$F^0(q,z) = e^{-q\|z\|}$	
**Confined**		Symmetric	Antisymmetric
	Inside	$F^{\mathrm{s}}_{\mathrm{i}}(q,z) = \sqrt{2}\cosh(qz)e^{-qh/2}$	$F^{\mathrm{a}}_{\mathrm{i}}(q,z) = \sqrt{2}\sinh(qz)e^{-qh/2}$
	Outside	$F^{\mathrm{s}}_{\mathrm{o}}(q,z) = \frac{1}{\sqrt{2}}e^{-q(\|z\|-h/2)}$	$F^{\mathrm{a}}_{\mathrm{o}}(q,z) = \frac{\mathrm{sign}(z)}{\sqrt{2}}e^{-q(\|z\|-h/2)}$

TABLE I. Weight functions used in the definitions of the surface and confined response functions (Eq. (**??**) and (**??**)).

channel. While our formalism allows us to treat the electric interactions of any static or dynamic distribution of charge, we restrict here to basic cases. We consider either an ion or a dipole in the center of the channel, as represented in Fig. 1a-b, to deduce the ion-ion and the dipole-dipole electrostatic interactions in presence of confinement.

To apply our theory and to compare with the existing literature, we need a simple model of the solid' surface response function. For this, we use the work of Kavokine *et al.* [2]. To start, let us duplicate their eye-opening derivation of the surface response function of an insulator of static dielectric constant $\epsilon_{\mathrm{m}} \approx 2$, taking into account the presence of water of static dielectric constant $\epsilon_{\mathrm{w}} \approx 80$ at the interface.

We consider an external electric potential $\phi_{\mathrm{ext}}(\mathbf{q}, z) = \phi^0_{\mathrm{ext}}(\mathbf{q})e^{qz}$ applied on the solid in $z < 0$. On the solid area the total electric potential is denoted $\phi_{\mathrm{m}}(\mathbf{q}, z) = \phi^0_{\mathrm{m}}(\mathbf{q})e^{qz}$ and the solid generates an induced electric potential $\phi_{\mathrm{ind}}(\mathbf{q}, z) = \phi^0_{\mathrm{m}}(\mathbf{q})e^{-qz}$ on the area $z > 0$. The

boundary conditions at $z = 0$ are the continuity of both the total electric potential ϕ and the electric displacement field $\mathbf{D} = -\epsilon\nabla\phi$, leading to

$$\phi^0_{\rm ext} + \phi^0_{\rm ind} = \phi^0_{\rm m} \tag{3}$$

$$\epsilon_{\rm w}\left(\phi^0_{\rm ext} - \phi^0_{\rm ind}\right) = \epsilon_{\rm m}\phi^0_{\rm m}. \tag{4}$$

Finally, we obtain the surface response function:

$$g^0_{\rm o}(\mathbf{q}) = \frac{\phi^0_{\rm ind}(\mathbf{q})}{\phi^0_{\rm ext}(\mathbf{q})} = \frac{\epsilon_{\rm m} - \epsilon_{\rm w}}{\epsilon_{\rm m} + \epsilon_{\rm w}} \tag{5}$$

This derivation is valid for an insulator, while the dielectric constant of the solid depends on the momentum q in general. A generalisation of this formula exists for a Thomas-Fermi model of solid, parametrised with a Thomas-Fermi wavelength $q_{\rm TF}$ [2]:

$$g^0_{\rm o}(\mathbf{q}) = \frac{\epsilon_{\rm m} f_{\rm TF}(q) - \epsilon_{\rm w}}{\epsilon_{\rm m} f_{\rm TF}(q) + \epsilon_{\rm w}}, \quad f_{\rm TF}(q) = \sqrt{1 + \frac{1}{\epsilon_{\rm m}}\left(\frac{q_{\rm TF}}{q}\right)^2}. \tag{6}$$

The case $q_{\rm TF} = 0$ corresponds to an insulator. The case $q_{\rm TF} \to \infty$, that is $g^0_{\rm o}(\mathbf{q}) \to 1$, corresponds to a metal. Let us highlight that $g^0_{\rm m}$ is negative at large wavelength and positive at small wavelength. The wavelength of the transition depends on the Thomas-Fermi wavelength. When the surface response function is negative, which is expected to happen at small distances, the effect of the boundary solids is to confine the electric field inside the channel: this is *interaction confinement*. At larger distances, the surface response function becomes ultimately positive: the electric field is no longer confined.

B. Interaction confinement for an ion and ion-ion electrostatic interaction

Let us start with the case of a static ion of charge e (see Fig. 1a). Such a charge generates a potential

$$\phi_{\rm ext}(\rho, z) = \int \frac{{\rm d}\mathbf{q}}{(2\pi)^2}\frac{e^2}{2\epsilon q}\sqrt{2}F^{\rm s}_{\rm o}(q,z)e^{i\rho\cdot\mathbf{q} - qh/2} \tag{7}$$

where we have introduced a Fourier decomposition into independent modes. Taking into account the effect of the material, the total potential in the inner space now writes

$$\phi(\mathbf{q}, z) = \frac{\sqrt{2}e^2}{2\epsilon q}\left[F^{\rm s}_{\rm o}(q,z) - g^{\rm s}_{\rm o}(\mathbf{q})F^{\rm s}_{\rm i}(q,z)\right]e^{-qh/2} \tag{8}$$

In particular, in the center of the channel, that is at $z = 0$, we obtain using Eq. (17) of the main text:

$$\phi(\mathbf{q}, z = 0) = \frac{e^2}{2\epsilon q}\left[1 - \frac{2g^0_{\rm o}(\mathbf{q})e^{-qh}}{1 + g^0_{\rm o}(\mathbf{q})e^{-qh}}\right] \tag{9}$$

consistently with the result of Kavokine *et al.* [2]. An ion in the center of the channel is a symmetric distribution of charge and then its potential is corrected by the symmetric response function of the outer medium. The effect of the medium is to screen the charge and then to confine the electric potential, as pictured in Fig. 1a.

Considering another ion of charge e in the center of the channel at a distance ρ, the ion-ion electrostatic interaction is

$$\mathcal{E}_{\rm ii} = \phi(\rho, z = 0) = \frac{e^2}{2\epsilon}\int_0^\infty \frac{{\rm d}q}{2\pi}J_0(q\rho)\left[1 - \frac{2g^0_{\rm o}(q)e^{-qh}}{1 + g^0_{\rm o}(q)e^{-qh}}\right] \tag{10}$$

where we have used isotropy and introduced the Bessel function of the first kind J_0. The first term provides te bulk interaction energy

$$\mathcal{E}^{\rm bulk}_{\rm ii}(\rho) = \frac{e^2}{4\pi\epsilon_0\epsilon_{\rm w}\rho} \tag{11}$$

while the second term provides a correction due to confinement. This correction can be computed numerically using Eq. (6). The resulting ion-ion interaction (normalised by $\mathcal{E}^{\rm bulk}_{\rm ii}(\rho)$) is plotted in Fig. 1c. Consistently with [2], we find that the interaction is reduced at large distance and increased at small distance for a generic solid.

C. Interaction confinement for a dipole and dipole-dipole electrostatic interaction

Let us now turn to an electrostatic dipole $\mathbf{d}$ oriented in the z direction (see Fig. 1b). Such a dipole generates a potential

$$\phi_{\rm ext}(\rho, z) = -\int \frac{{\rm d}\mathbf{q}}{(2\pi)^2}\frac{e^2 d}{2\epsilon}\sqrt{2}F^{\rm a}_{\rm o}(q,z)e^{i\rho\cdot\mathbf{q} - qh/2} \tag{12}$$

where we have introduced a Fourier decomposition into independent modes. Taking into account the effect of the material, the total potential in the inner space now writes

$$\phi(\mathbf{q}, z) = -\frac{\sqrt{2}e^2 d}{2\epsilon}\left[F^{\rm a}_{\rm o}(q,z) - g^{\rm a}_{\rm o}(\mathbf{q})F^{\rm a}_{\rm i}(q,z)\right]e^{-qh/2} \tag{13}$$

In particular, in the center of the channel, using Eq. (17) of the main text, the electric field writes

$$\mathbf{E}(\mathbf{q}, z = 0) = -\frac{e^2 d}{2\epsilon}q\mathbf{d}\left[1 + \frac{2g^0_{\rm o}(\mathbf{q})e^{-qh}}{1 - g^0_{\rm o}(\mathbf{q})e^{-qh}}\right] \tag{14}$$

An electrostatic dipole in the center of the channel is an antisymmetric distribution of charge and then its potential is corrected by the antisymmetric response function of the outer medium. Here again, the effect of the outer medium is to confine the electric potential, as pictured in Fig. 1b.

Considering another dipole of same moment $\mathbf{d}$ in the center of the channel at a distance ρ, the dipole-dipole electrostatic interaction is

$$\mathcal{E}_{\rm dd} = -\mathbf{d}\cdot\mathbf{E}(\rho, z=0) = \frac{e^2 d^2}{2\epsilon}\int_0^\infty \frac{\mathrm{d}q}{2\pi} q^2 J_0(q\rho)\left[1+\frac{2g_{\rm o}^0(q)e^{-qh}}{1-g_{\rm o}^0(q)e^{-qh}}\right] \tag{15}$$

The first term provides te bulk interaction energy

$$\mathcal{E}_{\rm dd}^{\rm bulk}(\rho) = \frac{e^2 d^2}{4\pi\epsilon_0\epsilon_{\rm w}\rho^3} \tag{16}$$

while the second term provides a correction due to confinement. This correction can be computed numerically using Eq. (6). The resulting dipole-dipole interaction (normalised by $\mathcal{E}_{\rm dd}^{\rm bulk}(\rho)$) is plotted in Fig. 1d. After a reduction of the interaction at small distances compared with the confinement h, the interaction energy is increased by a factor ≈ 2.6 independent on the model of solid.

To conclude this section, we have derived the correction of confinement to the electric potential. This approach generalises the results of interaction confinement [2] to the generic distribution of charge, or equivalently to a generic external potential, which we can decompose into a symmetric and an antisymmetric parts.

III. WATER RESPONSE FUNCTIONS AND SIMULATIONS

A. Numerical methods for water simulations

We have carried out both force-field (FF) and *ab initio* density functional theory-based (DFT) molecular dynamics (MD) simulations of water confined between two frozen graphene sheets for various separations h between the graphene sheets. For all systems, the number of water molecules confined between graphene sheets is held constant and chosen such as to reproduce the chemical potential of bulk water from SPC/E force field simulations according to [3]. FF-MD simulations are performed using GROMACS 2020 using the SPC/E water model and GROMOS 53A6 parameters for carbon atoms [4, 5]. Simulations are performed in the NVT ensemble using the CSVR thermostat at 300 K. We use a 2 fs time step and truncate Lennard-Jones interactions at 9 Å. Electrostatics employ a real-space cut-off at 0.9 nm while long-range interactions are handled with the particle mesh Ewald method. The considered systems have lateral dimensions of 42.6 Å × 44.3 Å and contain 210, 1872 and 3489 water molecules at h =7 Å, 34 Å and 60 Ågraphene sheet separations, respectively. After 1 ns of equilibration, we perform production runs of 20 ns.

DFT-MD simulations are carried out with the CP2K 6.1 software which performs Born-Oppenheimer MD [6]. Electronic structures are calculated at every time step based on the BLYP exchange correlation functional with Grimme-D3 dispersion correction [7], while core electrons are represented by GTH pseudo potentials and valence electrons are expanded in the DZVP-SR-MOLOPT basis set using a plane wave cutoff of 400 Ry [8, 9]. DFT-MD simulations are performed in the NVT ensemble at 300K with a 0.5 fs time step using the CSVR thermostat. The simulated systems have lateral dimensions of 29.82 Å × 27.05 Å and contain 90 and 420 water molecules for graphene sheet separations of h =7 and 17.8 Å, respectively. After preequilibration in FF-MD, DFT-simulations are equilibrated for another 5 ps followed by production runs of 70 ps.

B. Fluctuation-dissipation theorem

Our goal is to compute the surface response function defined in Eq. (8) of the main text as

$$g(\mathbf{q},\omega) = -\frac{e^2}{2\epsilon_0 q}\int \mathrm{d}z\mathrm{d}z'\, F(q,z)\chi(z,z',\mathbf{q},\omega)F(q,z'). \tag{17}$$

Using the fluctuation-dissipation theorem we then deduce

$$\mathrm{Im}\left[g_{\rm w}[F](\mathbf{q},\omega)\right] = \frac{e^2}{4\epsilon_0 q}\frac{\omega}{T}S[F](\mathbf{q},\omega) \tag{18}$$

where

$$S[F](\mathbf{q},\omega) = \int_{-\infty}^{\infty}\langle\delta n_{\rm w}[F](\mathbf{q},t)\delta n_{\rm w}[F](-\mathbf{q},t)\rangle e^{i\omega t}\mathrm{d}t \tag{19}$$

is a modified structure factor with en effective density

$$n_{\rm w}[F](\mathbf{q},t) = \int \mathrm{d}\mathbf{r}\, n_{\rm w}(\mathbf{r},t)F(q,z)e^{-i\mathbf{q}\cdot\mathbf{r}}. \tag{20}$$

The density $n_{\rm w}(\mathbf{r},t)$ is given by the simulation trajectory.

C. Evaluation of the time correlation function

For each time step, we first compute the Fourier-transformed charge density. From MD simulation, we have access to the center of each atom and use a partial charge $Z=\delta=0.41$e for the hydrogen atoms and $Z=-2\delta$ for the oxygen atoms. The density is then

$$n(\mathbf{q}) = \sum_j Z_j F(q,z_j)e^{iq_x x+iq_y y} \tag{21}$$

where F is the weight function and q_x, q_y are multiples of $2\pi/L_x$ ($2\pi/L_y$ respectively). We make this computation for $N_t = 10^5$ time steps, and then obtain the fluctuating part of the density by subtracting the average

$$\delta n(\mathbf{q},t) = n(\mathbf{q},t) - \langle n(\mathbf{q},t)\rangle_t \tag{22}$$

where $\langle A(t)\rangle_t = \frac{1}{N_t}\sum_t A(t)$.

We then carry out a fast Fourier transform (FFT):

$$\delta n(\mathbf{q},\omega) = \mathrm{FFT}[n(\mathbf{q},t)]_t \tag{23}$$

and only keep the positive frequencies. The response function is then computed as

$$\mathrm{Im}\,[g(\mathbf{q},\omega)] = \frac{e^2\omega}{4\epsilon_0 qT\mathcal{A}}|\delta n(\mathbf{q},\omega)|^2\frac{\mathrm{d}t}{N_t}. \tag{24}$$

where dt is the length of the time step. In practice, g only depends on the norm q. Finally, the spectra are smoothed by a Gaussian smoothing of width 0.2 meV for the FF simulations and 0.5 meV for DFT simulations.

D. Momentum dependence and position of the interface

The limited size of the simulation box does not allow us to access all momenta necessary for the computation of fluctuation-induced effects (typically below 1 Å^{-1}). Therefore, we restrict ourselves to one momentum compatible with the size of the box and extrapolate the response functions to lower momenta. We use $q_0 = 0.67$ Å^{-1} for FF simulations and $q_0 = 1$ Å^{-1} for DFT simulations.

From the simulations carried out with a large box in [1], we know that the non-confined water surface response function has only a weak momentum dependence: we therefore approximate the surface response function at all momenta by the one computed at q_0. For the confined response functions, however, the form of the weight functions imply that the static ($\omega = 0$) response fulfills $g_{\mathrm{w}}^{\mathrm{x}} \leq 1 + \eta e^{-qh}$, where $\eta = \pm 1$ for the symmetric (antisymmetric) component (see. ESI.1). In particular, the static antisymmetric response function goes to zero at $q \to 0$. We thus infer the momentum dependence of the confined response function according to

$$g_{\mathrm{w}}^{\mathrm{x}}(q,\omega) = \frac{1+\eta e^{-qh}}{1+\eta e^{-q_0 h}} g_{\mathrm{w}}^{\mathrm{x}}(q_0,\omega). \tag{25}$$

In practice, in the evaluation of friction coefficients, this procedure makes only a 1% difference compared to the assumption of no momentum dependence. Formally, however, such a procedure allows us to obtain well-behaved response functions that produce no unphysical overscreening.

The evaluation of the response functions requires to precisely define the positions of the interfaces at $z = \pm h/2$. In ref. [1], a procedure for positioning the interface based on imposing the compressibility sum rule for the surface response function was proposed. This procedure no longer formally holds in a confined geometry, but we expect the position of the interface to not be significantly affected by confinement. With our simulations at weakest confinement (FF at 60 Å and DFT at 34 Å) and following the procedure of ref. [1], we an effective distance between the plane of the carbon atoms and the water surface $d \approx 1.4$ Å, which is compatible with $d = 1.3$ Åfound in [1]. We then adopt the prescription $d = 1.4$ Å for the simulations under confinement.

[1] Kavokine, N., Bocquet, M.-L. & Bocquet, L. Fluctuation-induced quantum friction in nanoscale water flows. *Nature* **602**, 84–90 (2022).

[2] Kavokine, N., Robin, P. & Bocquet, L. Interaction confinement and electronic screening in two-dimensional nanofluidic channels. *The Journal of Chemical Physics* **157**, 114703 (2022).

[3] Loche, P., Ayaz, C., Wolde-Kidan, A., Schlaich, A. & Netz, R. R. Universal and Nonuniversal Aspects of Electrostatics in Aqueous Nanoconfinement. *The Journal of Physical Chemistry B* **124**, 4365–4371 (2020).

[4] Gromacs: High performance molecular simulations through multi-level parallelism from laptops to supercomputers. *SoftwareX* **1-2**, 19–25 (2015).

[5] Oostenbrink, C., Villa, A., Mark, A. E. & Van Gunsteren, W. F. A biomolecular force field based on the free enthalpy of hydration and solvation: The GROMOS force-field parameter sets 53A5 and 53A6. *Journal of Computational Chemistry* **25**, 1656–1676 (2004).

[6] Kühne, T. D. *et al.* Cp2k: An electronic structure and molecular dynamics software package -quickstep: Efficient and accurate electronic structure calculations (2020).

[7] Grimme, S., Antony, J., Ehrlich, S. & Krieg, H. A consistent and accurate ab initio parametrization of density functional dispersion correction (DFT-D) for the 94 elements H-Pu. *Journal of Chemical Physics* **132** (2010).

[8] Goedecker, S., Teter, M. & Hutter, J. Separable dual-space gaussian pseudopotentials. *Phys. Rev. B* **54**, 1703–1710 (1996).

[9] VandeVondele, J. & Hutter, J. Gaussian basis sets for accurate calculations on molecular systems in gas and condensed phases. *The Journal of Chemical Physics* **127**, 114105 (2007).
